# A Comprehensive Review on Silk Fibroin as a Persuasive Biomaterial for Bone Tissue Engineering

**DOI:** 10.3390/ijms24032660

**Published:** 2023-01-31

**Authors:** Minghui Li, Jiaqian You, Qiuyue Qin, Manxuan Liu, Yixin Yang, Kewen Jia, Yidi Zhang, Yanmin Zhou

**Affiliations:** 1 Jilin Provincial Key Laboratory of Tooth Development and Bone Remodeling, Hospital of Stomatology, Jilin University, Changchun 130021, China; 2 School of Stomatology, Jilin University, Changchun 130021, China

**Keywords:** silk fibroin, biomaterials, bone tissue engineering

## Abstract

Bone tissue engineering (BTE) utilizes a special mix of scaffolds, cells, and bioactive factors to regulate the microenvironment of bone regeneration and form a three-dimensional bone simulation structure to regenerate bone tissue. Silk fibroin (SF) is perhaps the most encouraging material for BTE given its tunable mechanical properties, controllable biodegradability, and excellent biocompatibility. Numerous studies have confirmed the significance of SF for stimulating bone formation. In this review, we start by introducing the structure and characteristics of SF. After that, the immunological mechanism of SF for osteogenesis is summarized, and various forms of SF biomaterials and the latest development prospects of SF in BTE are emphatically introduced. Biomaterials based on SF have great potential in bone tissue engineering, and this review will serve as a resource for future design and research.

## 1. Introduction

With increasing age, accidents, and orthopedic diseases, bone has gradually become the second most common tissue graft, and there is a growing demand for bone graft technology worldwide [1]. Bone defects could result in bodily dysfunction and thus have an impact on the life satisfaction of patients. Bone tissue has limited regenerative capacity, and its self-repair can occur only when the defect is small [2]. There are some limitations in autogenous bone and allogeneic bone transplantation, such as limited source, donor injury, immune rejection, etc. It is of wide clinical demand and practical significance to develop artificial bone materials for the effective healing and functional regeneration of lesions [3]. Bone tissue engineering (BTE) has been extensively developed, most recently, and is becoming a promising alternative for treating bone defects [4]. An in vivo-like microenvironment is created by combining cells and bioactive molecules with scaffolds. The ideal bone graft materials should have good biocompatibility, biodegradability, mechanical properties, and osteogenic properties to treat bone defects [5,6,7].

For several decades, silk fibroin (SF) has aroused growing interest. SF has been used as a suture in biomedicine for the past 20 years [8]. It has been demonstrated that SF is a potentially useful biomaterial in BTE because of its unique mechanical properties, controllable biodegradability, and good biocompatibility [9]. Various forms of SF-based biomaterials have been explored, such as films, particulates [10], hydrogels [11], sponges [12], fibers [13], and 3D porous scaffolds [14]. An immense number of studies have shown that SF combined with various organic and inorganic biomaterials, bioactive factors, and cell therapy could create an osteogenic microenvironment and further accelerate bone formation around bone defects [15,16,17,18].

Many published reviews have described the status of SF based materials for BTE [19,20,21,22,23,24]. Our purpose here is to broaden the understanding of the immunological mechanism of SF for osteogenesis and the latest research progress of SF biomaterials in BTE. This review starts with the introduction of the source, structure, and properties of SF, which is closely related to the application of BTE. Secondly, the various forms and different preparation methods of SF related to osteogenesis are summarized. This review seeks to highlight the immunological mechanism of SF, which may provide guidance for achieving better bone formation and enhancing their clinical translational potential. Based on recent advances and developments with SF-based materials for BTE, this review addresses the latest technology of SF blend bone substitutes. The summary of new bioactive materials and fabrication processing provides new insights and therapies for BTE. Finally, the challenges of SF-based bone tissue materials are discussed as the possibilities for future advancement.

## 2. Bone Tissue Engineering

BTE utilizes a special mix of scaffolds, cells, and bioactive factors to regulate the microenvironment of bone formation and form a three-dimensional bone simulation structure. Due to its good biocompatibility and biological activity, as well as the moderate mechanical properties of supporting cells, BTE is emerging as the most effective method for bone repair [6,25]. As BTE depends on our understanding of bone structure and composition, it is essential to have a basic grasp of bone biology. Bone is a special kind of connective tissue characterized as hard, dense, and highly vascularized. As an essential part of the human body, bone is essential for movement, support, structural integrity, and internal organ protection [26]. The bone structure and the main components, including minerals and extracellular matrix (ECM), affect the bone’s characteristics [27]. Cortical bone and cancellous bone are the two forms of mineralized tissues that are found in bone. Other components of bone involve bone marrow, endosteum, nerves, blood vessels, cartilage, and perichondrium [28]. Bone tissue consists of 65% inorganic matrix and 35% organic part [29]. The organic portion endows bone tissue flexibility and elasticity, while the inorganic portion provides strength and mechanical stress resistance [30]. It is well known that hydroxyapatite (HAP) is the major inorganic constituent. In the process of bone biomineralization, HAP are periodically deposited between collagen so that the mineralized fibril, non-collagenous proteins, and water are arranged in a complex hierarchical structure, finally forming the excellent mechanical properties of natural bone [31]. On the other hand, ECM can regulate cell attachment, growth, and transformation and induce the polarization of various progenitor cells and macrophages with good tissue remodeling properties [32]. Cell–material interactions are regulated by stimulating the ECM of bone on the surface of biomaterials [33]. Ideal tissue engineering scaffolds implanted into patients should be designed to mimic an ideal non-immune environment with natural three-dimensional structures and a variety of bioactive components [34]. To explore whether SF is a suitable material for BTE scaffolds, the biomaterial characteristics of SF will be discussed in the following paragraph.

## 3. Silk Fibroin: Source and Structure

SF can be derived from silkworm cocoons, spiders, scorpions, mites and flies, and so on [35,36,37]. The most common type of silk originates from the silkworm. Silkworm cocoons are widely raised worldwide to obtain silk [38]. The SF derived from silkworm cocoons is a mature textile fiber produced and processed at a rate of nearly 1000 metric tons per year, compared to spider fibers [39]. On the other hand, it is easy to operate and has good mechanical properties and high biocompatibility [40]. Recently, Zou et al. reviewed the effects of non-cocoon SF materials on the control of cell activity and tissue generation. According to the article, non-cocoon SF materials have a unique arginine-glycine-aspartic acid sequence to promote cell adhesion [41].

**Figure 1:**
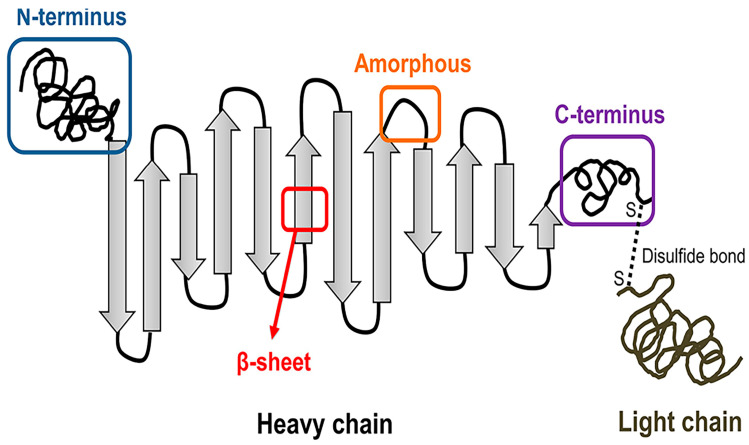
Structure diagram of silk fibroin (SF). The hydrophobic heavy (H-) chain and hydrophilic light (L-) chain are linked by disulfide bonds. Reproduced with permission from [47]. Copyright © 2018 American Chemical Society.

The primary constituents of SF are protein, a small number of lipids, and polysaccharides. SF has a considerable molecular weight modular hydrophobic structure that is interrupted by small hydrophilic groups. SF contains two major chains: the hydrophobic heavy (H-) chain and the hydrophilic light (L-) chain. These two chains are connected by disulfide bonds to construct H-L complexes (Figure 1) [42]. P25 is a hydrophobic glycoprotein linked to the H-L complex and plays an essential part in ensuring the structure’s integrity [43]. The amino acid sequence of the H-chain is mainly glycine. The Gly-X dipeptide sequences are repeated, accounting for 60–75% of the H-chain. Two hexapeptides, Gly-Ala-Gly-Ala-Gly-Ser and Gly-Ala-Gly-Ala-Gly-Tyr, comprise 70% of the Gly-X dipeptidyl sequence [22]. H-chain, L-chain, and P25 are blended in a 6:6:1 molar ratio [44]. In addition to the primary structure, the secondary and hierarchical structure of SF determines many of its biomaterial properties. The key crystalline structures of SF are silk I and II [45]. The hydrophobic domain of silk II, which consists of repeated amino acid sequences, is assembled into a β-sheet. Silk II is the state with the greatest degree of predictability considering the strong hydrogen bond interaction of β-sheet [46].

## 4. Silk Fibroin Dissolution Techniques

Silk fibers mainly comprise SF wrapped by sericin protein (SS) [48]. SF is composed of filamentous and semi-crystalline structural proteins. SS is a water-soluble gelling agent made of amorphous protein polymers [49]. The cocoon has to be degummed to remove SS, which, in association with SF, can enhance the adverse immune response [50].

**Figure 2:**
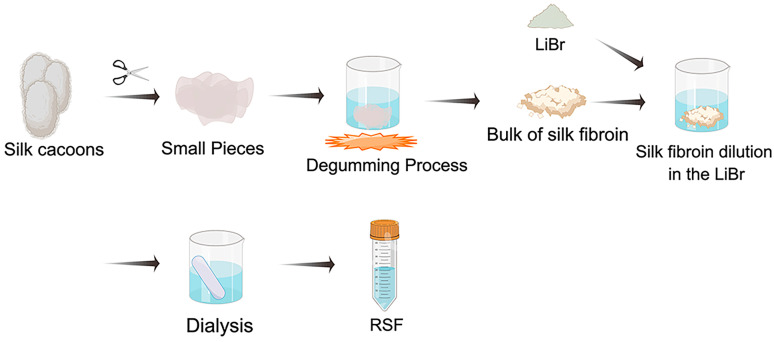
A schematical representation of obtaining regenerated silk fibroin (RSF) solution. Silk cocoons are cut into small pieces, which are purified from sericin by boiling them in degumming agents. The bulk of SF was obtained by drying overnight. RSF was formed by dissolving the bulk of SF in LiBr and then dialyzing.

After cutting silk cocoons into small pieces, the first step in obtaining regenerated silk fibroin (RSF) solution is degumming, which is usually carried out under boiling and degumming agents. Degumming agents mainly include alkaline, acid and neutral degumming agents, surfactants and enzymes [45]. The bulk of SF is obtained by drying overnight. LiBr solution, the widely used dissolving system, can guarantee a relatively high quality of SF in solution [51]. Therefore, degummed SF is usually dissolved in LiBr solution at 60 °C for 4 h. Dissolved fibroin solution was then dialyzed against the deionized (DI) water to finally obtain RSF (Figure 2) [52].

## 5. Properties of Silk Fibroin

### 5.1. Mechanical Properties

BTE scaffolds should be adapted to the mechanical properties of native bone and conduct appropriate loads. It has been previously reported that SF without SS exhibits better mechanical properties and facilitates the control of long-term conformation and stability [53]. The modulus of B. mori silk (with sericin) can reach 5–12 GPa, and the modulus of B. mori silk (without sericin) can reach 15–17 GPa [54]. Therefore, SF has great shear strength, tensile strength, and fracture resistance, making it an ideal material for bone construction [55]. However, SF scaffolds in BTE are mainly made from RSF solution. The structure and mechanical properties of SF can be changed due to the absence of compact hydrogen bonding at the inter-molecular level and the exposure of SF to extreme environments during the RSF preparation. Different treatments can provide various functions and mechanical properties [56,57]. In the current study, there are some cases of implantation failure owing to poor mechanical properties during the preparation phase [58]. The lack of a suitable secondary and hierarchical structure is to blame for this circumstance.

The mechanical strength of RSF can be effectively improved by controlling the secondary structure in the regeneration process [59]. Numerous research has shown that the crystallinity and stability of β-sheet secondary structures (silk II) can control the mechanistic qualities and characteristics of SF [60]. Temperature, pH, alcohol, ultrasonic treatment, and steam annealing could all influence the formation of β-sheet. In addition, methanol or ethanol treatment can increase glycine content in the amino acid sequence of SF protein and induce the formation of stable β-sheets [61]. Different modification strategies that address the above deficiencies, such as enzyme cross-linking, were developed, and SF composite scaffolds are beneficial for the improvement of mechanical properties. For example, Sheng et al. prepared enzyme cross-linked SF hydrogel enhanced by montmorillonite (MMT) nanoparticle with better mechanical properties and hydrophilicity than SF hydrogel. The compression modulus of SF-MMT nanocomposite hydrogel (24.78 ± 4.13 kPa) was markedly higher than that of SF hydrogel (16.77 ± 1.99 kPa, *p* < 0.001) [62]. Furthermore, strontium-substituted calcium silicate/SF composite materials were developed by Zhou and colleagues, which showed the same results as the previous study [63].

### 5.2. Biocompatibility

Biocompatibility can enable cells to adhere and migrate into scaffolds, which is an essential element for the success of BTE. SF was approved by the FDA in 1993 and used as a suture [64]. In vivo studies have demonstrated that SF is compatible with blood [65,66]. In 1995, Minoura et al. successfully cultured fibroblasts on SF-coated films [67]. In addition, the biocompatibility of SF has been the subject of several investigations. For example, Fan et al. used a large animal model, porcine anterior cruciate ligament (ACL), for evaluation in vitro and in vivo. Observations made using confocal microscopy showed that scaffolds supported both high cell growth and good cell survival. The cells were dispersed across the scaffolds with negligible cell loss [68]. A large number of reports have found that SF composite scaffolds are more biocompatible when applied to BTE. Jo et al. conducted an in vivo trial to evaluate the effect of alginate/HAP/SF composites as bone substitutes, and the results showed no infection and reduced immunogenicity for up to four weeks. The expression level of tumor necrosis factor-α (TNF-α) was significantly decreased, while the expression rates of Runx2 and fibroblast growth factor (FGF)-23 were higher in the tumor necrosis factor [69]. Other than that, the SF/gelatin microcarrier prepared by Luechford et al. improved cell adhesion and proliferation. After 28 days in the osteoblastic culture medium, the cells showed osteoblastic differentiation under the microscope stained with alkaline phosphatase (ALP) [70].

### 5.3. Biodegradability

Scaffolds for tissue engineering should be gradually replaced by cells and extracellular matrix, so the by-products of biodegradation must not be harmful and should not affect the functions of other tissues or organs [71]. Amino acids and peptides are the results of the degradation process which do not cause immunogenic reactions and are easily absorbed by the body [72]. However, the challenge is the degradation of β-sheets in SF, as previously proposed β-amyloid might participate in the development of Alzheimer’s disease [73]. Different techniques can be used in vivo and in vitro to identify biomaterial degradation. In vitro, the degradation of tested biomaterials can be assessed through mass loss and morphological changes [74]. In vivo, animal models were used for histological and fluorescence studies after implantation [75]. SF is an enzyme-degradable polymer. The degradation of SF is mediated by matrix metalloproteinase (MMP) and integrins expressed by osteoblasts and osteoclasts. The findings show that water-soluble silk I and insoluble silk II proportion have an impact on it. To be specific, higher silk I content silk films degrade more quickly than those with higher silk II content [76,77]. Alternatively, Protease XIV is derived from *streptomyces griseus.* It has long been considered as a model enzyme for examining the behavior of SF degradation and is the most commonly used enzyme for SF degradation [47].

Another challenge that currently exists is that the scaffolds require slow degradation and load-bearing capacity to maintain their mechanical properties for more extended periods in BTE [78]. However, controlling the time and degradation of SF remains a challenge. Umuhoza et al. summarized and reported that the regulation of the biodegradation rate of SF materials was related to the raw material status, scaffold preparation method, morphological characteristics, and host factors. For example, the crystal content in regenerated SF is inferior to those in native SF. Consequently, the degrading characteristics of natural and reconstituted SF might vary considerably. Besides that, degumming can alter the SF structure and, as a result, it greatly impacts the features of the finished product [79]. Biodegradability information about different morphological characteristics of SF has also been collected in vivo in small animal models, revealing that fibroin yarn and scaffold completely biodegrades in 18–36 months, while for a hydrogel, the biodegradation is completed in 12 weeks. In addition, fibroin nanofibers completely biodegrade in 8 weeks [78,80,81,82].

## 6. Silk Fibroin for Osteogenesis by Immunoregulation

**Figure 3:**
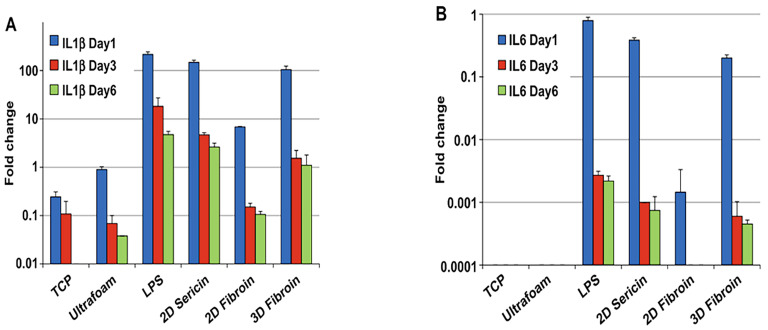
Monocyte responsiveness to silk-based biomaterials with different physic-chemical characteristics: cytokine gene expression. (**A**) Gene expression of IL-1β. (**B**) Gene expression of IL-6. Reproduced with permission from [83]. Copyright © 2013 Published by Elsevier Ltd.

With bone immunology playing an increasingly important role in bone tissue engineering in recent years, the role of SF as commonly used biomaterials in the induction of macrophage polarization has been gradually explored. The first inflammatory phase is essential for the completion of bone repair, as SF could induce the M1 phenotype in macrophages. As inflammation progresses, the pro-inflammatory M1 phenotype is polarized into the anti-inflammatory tissue repair M2 phenotype. Following that, the interaction between macrophages and osteoblasts may substantially promote bone repair. Bhattacharjee and colleagues co-cultured freshly purified peripheral blood monocytes with lyophilized 3D fibroin scaffold and observed gene expression of IL-1β (Figure 3A) and IL-6 (Figure 3B). Higher gene expression of IL-1β and IL-6 was detectable in cells stimulated with 3D fibroin group on the first day, which are responsible for the initiation of inflammatory response. However, gene expression of IL-1β and IL-6 significantly decreased after six days. The study also found that different immunogenicity of different SF materials is attributed to the physical characteristics and protein conformation of the materials, which may be influenced by the content of the β-sheet in SF [83]. In addition, it was reported that when SF nanoparticles were fixed on the surface of titanium, the expression of CD86 in the Ti-SF groups decreased seven days after surgery compared with the Ti group. However, CD206 expression was greater in the Ti-SF groups. At the same time, the expression of collagen (COL) 1, osteopontin (OPN), and new bone area were more elevated in Ti-SF [84]. Another observation was made by immobilizing non-mulberry SF on the titanium surface to study the effect on osteoblast-macrophages. After 24 h, the protein-coated surfaces exhibit a reduced inflammatory response as measured by TNF-α and IL-1β released by macrophages. The proliferation of macrophages is confirmed by the increased generation of nitric oxide (NO) from mono- and co-cultures on all Ti surfaces on days 1 and 3. Intriguingly, the direct co-culture model of macrophage and osteoblast produces significantly less TNF-α and IL-1β and NO production, which can be explained by the cellular cross-talk [85].

## 7. Silk Fibroin Processing Methods

**Figure 4:**
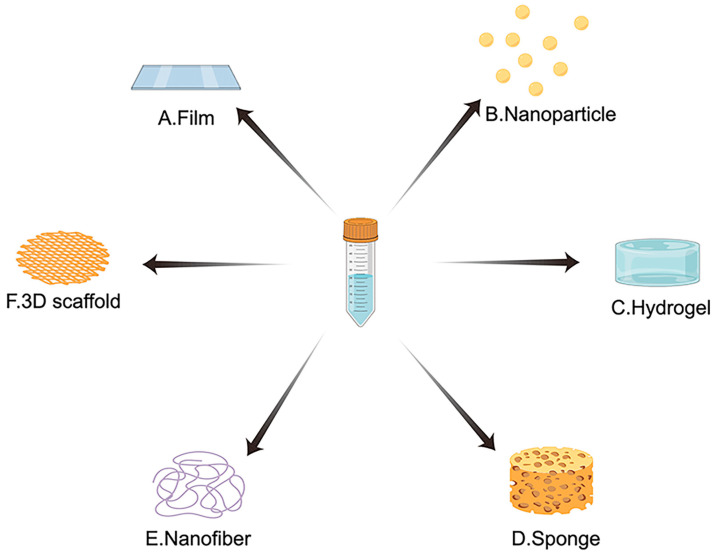
Multiple typical forms of SF as a functional biomaterial for bone tissue engineering (BTE): (**A**) Film; (**B**) Nanoparticle; (**C**) Hydrogel; (**D**) Sponge; (**E**) Nanofiber; (**F**) 3D-printed scaffold.

SF scaffolds can be fabricated into various forms for BTE using different techniques: films, nanoparticles, hydrogels, sponges, nanofibers, and 3D-printed scaffolds (Figure 4).

### 7.1. Films

SF films are prepared by adding aqueous, acidic, and ionic SF solutions to the substrate and then drying the solution [86]. The main techniques include spin coating and vertical deposition. Spin coating alternates the regenerated SF solution and ethanol on the surface. Vertical deposition immerses dry glass into regenerated SF solution, which was then dried at 50 degrees [87]. On the other hand, controlling the surface qualities of films through photolithography and sophisticated printing techniques is critical for directing and increasing cell adherence and development [88]. To improve the stability of the film, temperature-controlled water vapor annealing (TCWVA), stretching, ethanol, and controlling drying were used to induce and increase the content of β-sheets and prevent the film from dissolving in water [76,89]. Wang et al. first modified magnesium alloy by vacuum UV ozone surface activation method and prepared magnesium alloy coated with SF, which provided more possibilities for bone implantation [90].

### 7.2. Nanoparticles

SF nanoparticles can be prepared from SF solutions by the following methods: freeze-drying, grinding, spray-crushing, spray-drying, self-assembly, and freeze-thawing [91,92]. The desolvation and salting-out process is the most commonly used method to prepare SF nanoparticles due to comparatively mild conditions and simplicity of operation. However, organic solvent and salting-out agents residue will be present in both methods, respectively [10,93]. Considering their high surface-to-volume ratio, superior solubility, and outstanding chemical modification capabilities, nanoparticles have attracted more and more attention [94]. SF nanoparticles are especially used as carriers for the delivery of drugs and bioactive factors [95]. For example, Subia et al. bound folic acid to SF to enhance drug loading, targeting, and controlled release [96]. The functionalized SF-based nanoparticles can be designed to improve the therapeutic efficiency of drugs encapsulated into these nanoparticles. The introduction of different surface modifications brings many optimized and new functionalities to SF nanoparticles [97,98,99,100,101,102]. The preparation of fine and uniform SF nanoparticles remains a challenge. There are benefits and drawbacks associated with each strategy, and it is imperative to select a suitable approach for forming SF-based nanoparticles for BTE.

### 7.3. Hydrogels

The solution–gel transformation of the SF aqueous solution forms SF hydrogels. One method is physical cross-linking through eddy current, ultrasound, change of pH or temperature, irradiation, freezing, and electromagnetic treatment [103,104]. Sonication is a crucial new method for initiating fast sol–gel transitions. Three-dimensional viscoelastic polymer networks known as hydrogels may diffuse molecules and cells [105]. The microenvironment is similar to natural tissues and can promote the delivery of nutrients and cytokines [106]. Consequently, SF hydrogels can serve as a medium for encapsulating cells. For example, Wang et al. entrapped human marrow mesenchymal stem cells (MSCs) into ultrasound-induced RSF hydrogels, and cells continued to be alive and multiply while being maintained under circumstances of static culture for several weeks [107]. Another method is chemical cross-linking, in which horseradish peroxidase (HRP) is used for enzymatic cross-linking in the presence of hydrogen peroxide (H_2_O_2_) to form SF hydrogel [108]. After going through the gelation process, the formation of the SF aqueous solution will shift from the random coil structure (silk I) to the β-sheet structure (silk II) [109]. Compared with chemical cross-linking, the gel process of physical cross-linking is slower, but it has the advantage of creating a more uniform β-sheet form, which is a better cross-linking form [46]. Accelerating the formation of SF hydrogels requires raising the protein content, the temperature, and the incorporation of calcium ions [110]. Moreover, the functional design of SF has attracted worldwide attention, including high strength, injectability, healing, adhesion, conductivity, environmental responsiveness, and 3D printing [11]. The high-strength SF hydrogel can be prepared by physical cross-linking, double cross-linking, double network, and composite hydrogels [46]. It has been reported that a novel form of photo-crosslinked interpenetrating polymer network hydrogel has been demonstrated to deliver drugs [111].

### 7.4. Sponges

SF sponges are interconnected porous structures with high porosity, excellent mechanical characteristics, and biodegradability, which can encourage cell adhesion, proliferation, and migration [112]. SF sponges can be produced in various ways. For example, the regenerated SF solution sublimates ice crystals and leaves pores by freeze-drying, forming soft, porous sponges with a bone lamellar-like structure. By changing the RSF solution’s pH, concentration, and freeze-drying temperature, the porosity and pore size of the SF sponge may be modified. Ice templating is a recently developed method for the adequate preparation of porous sponges, and many studies have shown that it can form a more optimized pore structure than conventional freezing. Moreover, it has the advantage of being simple, fast, and convenient. Wang et al. prepared an SF/nano-hydroxyapatite/graphene oxide scaffold with a directional channel structure by directional freezing. This structure is more favorable for migration and bone formation, and for differentiation of BMSCs [113]. The same results can be seen in ZnSr-doped β-TCP/SF scaffolds [114]. Font Tellado S and colleagues designed biphasic SF scaffolds with two different pore arrangements and mechanical strengths by a combination of targeted freezing and freeze-drying for tendon/ligament-BTE [115]. In addition, using the pore-forming agent and the gas-foaming manufacturing process, SF porous scaffolds with varying pore diameters may be manufactured [116].

### 7.5. Nanofibers

SF fibers are produced by a variety of flexible methods and can be formed by electrospinning [117], wet spinning [118], dry spinning [119], and other technologies. The most common is electrospinning, in which droplets are electrified to produce a jet, and when the jet is stretched to a thinner diameter, the fibers are rapidly cured and deposited [120]. Electrospinning SF nanofiber mats have a high surface area and a porous structure, both of which offer favorable conditions for cell inoculation, adhesion, and proliferation [121]. The concentration of SF is a significant component that influences the morphology and porosity of the nanofiber networks [122]. Mao et al. coated electrospinning poly (L-Lactic acid) (PLLA) fibers with SF nanofibers, which may improve cell adherence and accelerate the growth of protrusions [123]. In addition, doping graphene oxide (GO), containing silver and magnesium ions [124] and adding polyethylene oxide (PEO) nanoparticles [125], can enhance the antibacterial properties of regenerated SF mats and resist infections that may develop during the osteogenesis process. However, electrospinning still has some limitations, which can be combined with other manufacturing methods to compensate. For example, the combination of electrospinning and freeze-drying can promote the delivery of growth factors, increase the stimulation of the bone microenvironment, and enhance osteogenic differentiation [126].

### 7.6. 3D-Printed Scaffolds

3D porous SF scaffolds have excellent porosity and can simulate the biological microenvironment in vivo, which is beneficial to the adhesion, growth, migration of cells, and the transfer of nutrients and metabolic wastes [127]. In addition, 3D printing allows cells to be encapsulated in hydrogels to form a natural tissue-like environment [128]. There is no doubt that SF hydrogel is an ideal and attractive choice for 3D printing. However, the literature still reports limited examples, as many challenges need to be overcome [129]. The critical characteristics of bioink suitable for 3D printing are that it is cell-friendly, reproducible, and has physical chemical gradients suitable for printing [130]. However, the viscosity of SF hydrogel is higher and a larger pressure is needed for the extrusion of bioink from the small nozzle, which can cause the nozzle to be blocked and cell death [131]. This is due to the fact that silk I is a metastable state and tends to aggregate and form β-sheet structures [132].

In order to be suitable for 3D printing, the excellent rheology is the basic requirement for bioink, so SF hydrogels usually consist of SF with additives [133]. Furthermore, effective crosslinking method should be adopted to improve cell viability [134]. In BTE, a variety of methods, including those that make use of enzymatically cross-linked SF hydrogels, have been investigated. According to a study by Costa et al., there is a potential for printing silk scaffolds utilizing enzymatic methods, which have good mechanical properties, controllable biodegradability, and adjustable pore structure and shape. In addition, it has unique features of shape memory, which can open up the possibility for personalized implantation of bone defects [135]. Furthermore, digital light processing (DLP) bioprinting is a light-assisted direct approach that can circumvent some of the most significant limitations of inkjet and extrusion bioprinting. So far, several investigations have been completed utilizing methacrylated silk fibroin (Sil-MA) as a material for DLP bioprinting. Experimental tests have proven that Sil-MA has outstanding mechanical and rheological properties, excellent structural stability, and good cell compatibility [136]. 3D printing hydrogels of Sil-MA exhibit bone-mimetic structures and compressive modulus ranging from ≈12 kPa to ≈96 kPa. Encapsulation of pre-osteoblasts using 3D bioprinting Sil-MA hydrogels was effective, resulting in excellent viability of the cells. It was shown that cell proliferation and morphology are good. Subsequently, calcium deposition is enhanced, demonstrating that the hydrogels that encapsulate cells can promote the ability of osteogenesis [137]. Recently, fluorescent SF bioink was made into glycidyl methacrylated fluorescent SF (FSGMA), which suggested a potent tool for encapsulated cell tracking and observing real-time degradation of the hydrogels [138]. This study provided fuller data support for the further application of SF in 3D printing.

## 8. Application of Silk Fibroin Biomaterials for BTE

### 8.1. SF-Based Scaffolds for BTE Applications

The optimal scaffolds for BTE should be porous and biocompatible in order to promote cell attachment, growth, differentiation, and migration. On top of that, they must have sufficient toughness and biodegradability. Bone tissue engineering requires careful consideration of the materials used and the structures designed. SF is a reasonable choice for BTE because of its good properties and the ability to support osteogenesis and oxygen transport. For instance, COL-1 expression was observed on scaffolds cocultured with human amniotic mesenchymal stem cells (hAMSCs). The scaffolds stimulated the growth of hAMSCs and elevated their COL-1 expression. In vitro, the scaffolds significantly increased ALP activity and bone mineralization, and elevated the expression of osteogenic-related proteins. On the other hand, the scaffolds facilitated the angiogenic differentiation of hAMSCs. The cocultured scaffolds accelerated bone growth in the treatment of severe mouse calvarial defects [139]. In addition, combining SF with other materials could increase the roughness of the material surface, enhance cell adhesion and promote osteogenic properties. The latest progress of SF-based scaffolds as bone construction is summarized in Table 1. Chen et al. prepared composite scaffolds through incorporating SF nanoparticles into PLLA, which was beneficial in promoting surface roughness and albumin attachment. Furthermore, SF/PLLA scaffolds were proven to be able to facilitate an increase in mouse osteoblastic (MC3T3-E1) cells’ osteogenic differentiation [140].

A recent study showed that the mechanical properties of SF hydrogel-derived scaffolds can meet the load-bearing requirements of bone regeneration. Kim et al. used γ-ray irradiation technology to prepare SF composite hydrogel containing hydroxyapatite nanoparticles. The findings revealed that the composite hydrogel could improve the mechanical properties of hydrogel and effectively stimulate the functional activity of hMSCs and induce bone regeneration [141]. At the same time, a portion of studies suggested that carbonate hydroxyapatite (CHA) appears to have more potential than HAP as a re-substitute for bone scaffold development to enhance bone regeneration. When macrophages are co-cultured with polycaprolactone/SF/carbonated hydroxyapatite scaffolds prepared by electrostatic spinning technique, the scaffolds are found to promote macrophage polarization toward M2 via the JAK/STAT5 pathway, leading to the bone microenvironment to promote osteoblast differentiation. The PCL/SF/CHA group showed signs of being more successful than the other groups in vivo in stimulating bone repair in cranial lesions [142]. Recent evidence from another study by Del Bianco et al. suggested that SF membranes containing magnetic nanoparticles triggered a kind of benign magneto-mechanical stimulation conducive to osteogenic differentiation under the applied magnetic field, which provides further theoretical support for the application of SF in BTE [143].

**Table 1:** SF-based scaffolds for BTE applications.

Material	Processing Method	Cell	Osteogenic Effect	Ref.
SF/PCL/CHA	Electrospinning	BMSCs	Activation of the JAK/STAT5 pathway led to the creation of a pro-osteogenic milieu, which facilitated the differentiation of osteoblasts. This was accomplished by shifting the polarization of macrophages toward M2.	[142]
SF	Digital light processing	Mouse osteoblastic cells (MC3T3-E1)	Effectively promoted cell proliferation, resulting in favorable cell shape and cytoskeletal morphology, and led to enhanced calcium deposition over a period of up to 14 days.	[137]
SF/apatite	Unidirectional freeze-drying	BMSCs	Exhibited high cytocompatibility and considerably enhanced bone formation; these results were seen in vitro and in vivo.	[17]
SF/PDA/E7	Electrospinning	BMSCs	Creating osteoinduction conditions improved BMSCs’ biocompatibility, stimulated cell proliferation and adhesion, and enhanced their osteogenic differentiation.	[144]
PCL/Fe-BG hMSCs/SF-PVP-nHA	3D bioprinting	hMSCs	Increased the levels of osteogenic markers and aided the development of osteogenic-primed MSCs cultured in encapsulation.	[145]
SF/HAP/GPM	Freeze-drying	/	Inhibited miR-214 expression in MC3T3-E1 in vitro, which in turn enhanced the expression levels of activating transcription factor 4, therefore promoting activity of osteoblasts, extending the expression levels of osteogenic genes and proteins, and enhancing osteogenic differentiation.	[146]

### 8.2. SF as a Scaffold for Growth Factor Delivery

BTE simulates the hierarchical structure of bone tissue by designing growth factors into biomaterials with enhanced bone-inducing abilities. Many studies have demonstrated that growth factor composite scaffolds are more effective in local delivery and bone formation. Bone morphogenetic protein-2 (BMP-2), as a multifunctional paracrine growth factor, is a member of the TGF-β superfamily and is crucial in the mechanism of osteoblast differentiation and bone formation [147]. When BMP-2-loaded absorbable SF screws were implanted into the distal femur of rats, osteoclasts and osteoblasts were recruited more, allowing more collagen and osteoid deposition than unloaded screws [148]. In addition, SF as a carrier can control the release of growth factors, which is also essential in promoting bone regeneration [149]. Shi and colleagues prepared SF nanoparticles containing BMP-2 with an average size of about 250 nm. BMP-2 was released in a regulated manner. Utilization of these compounds increases ALP activity, osteogenic gene expression, and osteogenic differentiation of MSCs [150]. Bessa et al. reported that SF microparticles prepared by the mild method were used as carriers for the transfer of BMP-2, BMP-9, or BMP-14. The release kinetics suggest that BMP was released in two stages, with a burst of release in the first two days, followed by a slow release lasting 14 days. This allows BMPs to target specific tissues and has the advantage of continuous or controlled release, improving the bioavailability of growth factors [151]. However, an increasing number of adverse events associated with the clinical application of rhBMP-2, such as ectopic bone formation, inflammation and bone resorption, and uncomfortable lipogenesis, are reported [152]. Most complications are thought to be due to excessive superphysiological concentrations of rhBMP-2. Noggin is the main antagonist of BMP-2. siNoggin-transfected MC3T3-E1 cells were used to enhance the osteogenic effect of BMP-2 dose reduction in 3D SF scaffolds. The outcomes demonstrated that siNoggin decreased Nog gene expression, but osteocalcin (OCN) gene expression was five times higher in the siNoggin group compared to the control group. Histological staining revealed that the siNoggin group had significantly more mineralized regions than the control group. This non-gene integration strategy has the potential to enhance the safety of tissue regeneration therapy [153].

Vascularization is also crucial in bone regeneration. Vascularization in bone defects affects the action of cells and signaling molecules involved in bone regeneration [154]. A functional vascular network can provide oxygen and nutrients, regulating the proportionality of osteoblasts and osteoclasts to stimulate the release of VEGF and induce osteoblast differentiation [155]. SF nanoparticles loaded with VEGF helped to maintain the release of VEGF [156]. VEGF was loaded onto the SF nanoparticles and embedded into the silk scaffold containing vancomycin to form a dual drug delivery system. The results suggested that the constructed co-delivery system can deliver antibiotics and angiogenic factors, which might be a possible application to treat contaminated bone injury [157].

It is known that SF hydrogel combined with VEGF and BMP-2 can promote the formation of the blood vessel and bone. Bai et al. created a novel kind of hydrogel, capable of multiple transitions between hydrogel and dissolution, which is helpful for the injectable drug delivery system [158]. Furthermore, Zhang et al. prepared injectable ultrasound-induced silk hydrogel to deliver VEGF (165) and BMP-2 for the maxillary sinus floor elevation. The findings demonstrated that injectable SF hydrogels could be utilized to administer various growth factors to irregular bone voids in a less invasive way [159]. Binding BMP-2 and VEGF to SF microspheres was subsequently incorporated into SF/nHAP scaffolds for regulated release. Early bone repair is associated with the quick initial release of VEGF and encouragement of angiogenesis, followed by the comparatively slow and continuous release of BMP-2 for osteogenic differentiation [160]. This research suggests a viable strategy for the continuous delivery of growth factors for use in BTE.

### 8.3. SF as a Scaffold for Stem Cell-Based BTE

Stem cells possess the capacity for multi-directional differentiation, and BMSCs are often used in BTE to repair bone regeneration effectively [161]. The osteogenic differentiation of stem cells results from precise regulation of a large number of gene activations/silencing. Among them, PHF8 plays an essential role in the fibroin scaffold filled with BMSCs in osteoblast differentiation and skull regeneration after implantation of mouse skull defects. PHF8 is a major H4k20/H3k9 demethylase which can epigenetically regulate the activity of unique SAT-rich sequence-binding protein 2 (SATB2). SATB2 triggers the osteoblastic differentiation of BMSC and induced pluripotent stem cells (iPSCs) and upregulates the expression of Runx2 and bone matrix proteins by inhibiting HoxA2 and enhancing the function of osteoblast-determinant Runx2, BSP, and OCN [162,163].

According to the findings of studies, the osteogenic differentiation of stem cells can be affected by adjusting the properties of the SF scaffold. Various forms of SF scaffolds have been used as substrates for loading BMSC, such as electrospinning SF mats supporting the adhesion, diffusion, and growth of BMSC in vitro [164]. Subsequently, electrospinning SF scaffolds modified with polydopamine (PDA), grafted with E7, were prepared to improve growth, adhesion, and osteogenic differentiation of BMSC. This research proves that the composite scaffolds possess the capability to recruit BMSCs and accelerate bone formation, which is associated with the SDF-1 α/CXCR4 axis and AKT, ERK, and p38 signaling pathways [144]. Studies have shown that the scaffold stiffness might affect the three-dimensional differentiation ability of MSCs in vitro. Rockwood et al. implanted MSCs into an SF composite scaffold reinforced with porous SF particles and found that the osteogenic ability of MSCs was gotten better. This provided a means to improve the osteogenic outcome [165]. Additionally, the osteogenesis of BMSC is dependent on the scaffold’s pore size. Three kinds of fibronectin/gelatin-coated SF scaffolds with large, medium, and small/average pore sizes were prepared by the salt leaching method. The fibronectin/gelatin-coated SF scaffolds with an average pore size of 173.8μm were most suitable for osteogenic differentiation of BMSC in vitro [166].

Recently, a novel “sandwich” approach has been discovered. SF/nanohydroxyapatite bio-inks loaded with MSCS are bio-printed together with thermoplastic inks formed by paramagnetic iron-doped bioactive glass-polycaprolactone blends for bone fracture treatment. In this study, pulsed magnetic field drive positively affects the osteogenesis and maturation of bioprinting structures through mechanical conduction, which provides SF as a scaffold for stem cell-based BTE with a new and viable option [145]. Furthermore, when co-cultured with MSCs, RSF united with growth factors can enhance cell adhesion and osteogenic differentiation, stimulate ALP activity, and promote bone formation in vivo [167]. Karageorgiou et al. reported that SF scaffolds packed with BMP-2 and implanted with MSCs had increased ALP activity, calcium deposition, and transcription levels of bone sialoprotein, osteopontin, osteocalcin, and osteogenic marker gene cbfa-1. There was higher bone formation in comparison to control without BMP-2 supplementation [168].

**Figure 5:**
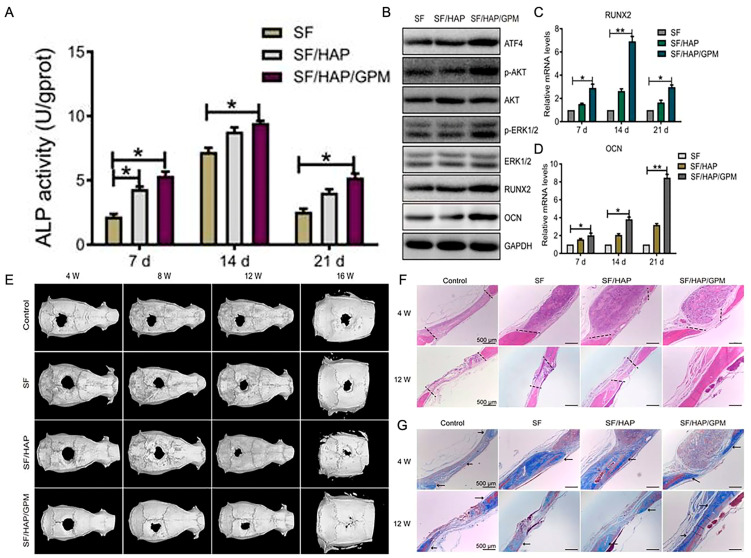
The capacity of SF/HAP/GPM scaffolds for osteogenesis. (**A**) The ALP activities of MC3T3-E1 cells on SF-based scaffolds for 7, 14, or 21 days. (**B**) The expression levels of ATF4, p-Akt, Akt, p-ERK1/2, ERK1/2, RUNX2, and OCN in MC3T3-E1 cells cultured on SF, SF/HAP, and SF/HAP/GPM scaffolds for 14 days. (**C**,**D**) The expression levels of RUNX2 and OCN on SF, SF/HAP, and SF/HAP/GPM scaffolds were measured by qRT-PCR. (**E**) Reconstructed micro-CT images of calvarial deficiencies following implantation of SF, SF/HAP, and SF/HAP/GPM scaffolds for 4, 8, 12 and 16 weeks. (**F**,**G**) Histological sections of control, SF, SF/HAP, and SF/HAP/GPM scaffolds were stained with H&E and Masson’s trichrome after 4 and 12 weeks of implantation. * *p* < 0.05 and ** *p* < 0.01. Reproduced with permission from [146]. Copyright © 2019 Ivyspring International Publisher.

Exosomal miRNAs derived from BMSC are crucial for bone formation and resorption [169]. Ou et al. assembled PEI-GO complexes loaded with miR-214 inhibitors into SF/HAP scaffolds to fix critical-size bone deficiencies in rats without loading osteoblasts. The results showed that the scaffolds have strong mechanical resistance. The structure promotes cell adhesion and proliferation and can control the release of miR-214 inhibitors. By inhibiting the expression of miR-214 and inversely increasing the expression of activated transcription factor 4 (ATF4), the AKT and ERK1/2 signaling pathways in MC3T3-E1 were activated to achieve the osteogenic activity of endogenous osteoblasts. The capacity of SF/HAP/GPM scaffolds for osteogenesis was observed (Figure 5) [146]. miR-23a-3p is highly expressed in the exons of BMSCs and can target interferon regulatory factors 1(IRF1). It can facilitate the conversion of M1 to M2, reduce the early inflammatory reaction, and contribute to the early repair of bone tissue [170].

With the gradual increase in the research on cellular exosomes in recent years, the significance of exosomes in the stimulation of bone regrowth has been demonstrated in a number of investigations [171]. However, the delivery of exosomes is still a significant challenge. Sun and colleagues prepared a new type of SF sponge by low-temperature freezing self-assembly, which serves as a platform for delivering enzymes to respond to the biodegradation of exosomes. The silk sponge produced by this method has the formation of Silk I structure, which degrades more quickly. This encapsulation realizes the continuous release of exons and the maintenance of biological activity. The SF sponges with and without exosomes were implanted subcutaneously in nude mice. It was found that the sponge group containing exosomes showed better angiogenesis and tissue inward growth effects [172]. The ongoing in-depth studies of miRNAs derived from BMSC provide new insights and therapies for BTE.

## 9. Conclusions and Perspectives

BTE plays an extremely significant role in treating bone abnormalities and can meet urgent clinical needs. In order to restore the function of the system, the tissue must coordinate with the human immune system without adverse effects. A better understanding is needed regarding SF for osteogenesis by immunoregulation. This review may provide guidance for achieving better bone formation. Further, it helps to understand the immune response of SF materials in vivo and enhance their clinical translational potential. SF has excellent mechanical properties, biocompatibility, and an easily controlled degradation rate, making it a unique polymeric biomaterial for guided bone regeneration. SF has been taken on a variety of shapes, such as films, nanoparticles, hydrogels, sponges, fibers, 3D scaffolds, etc. It has a broad application prospect in the biomedical field, and many studies have used it for bone tissue repair and functional recovery. In addition, SF can combine with different biomaterials to form composite scaffolds, which can improve the performance of scaffolds and promote the formation and mineralization of new bone, enhancing the applicability and new possibilities of SF in the field of BTE.

However, there are still certain issues that need to be addressed and resolved in SF-based biomaterials. For example, the mechanical characteristics of SF scaffolds are relatively poor, and their clinical applications and systematic research need further study. SF is a non-autogenous biomaterial, and there are still some adverse immune responses, which may be due to residual sericin. Then again, the degradation products of SF biomaterials may trigger the immune system. Therefore, a great deal of work is required to be done to elucidate the long-term safety of SF scaffolds further. Although it has been shown that SF material has achieved exciting bone regeneration in small animal models, it is still lacking in large animal models. Furthermore, research on the osteogenesis signal pathway of SF needs to be more thorough. So far, SF-based biomaterials have not entered the phase of human trials yet, and the clinical translation may still take time. There is still much work to be done to accelerate the development of SF-based products for BTE, and an increase in the usage of SF scaffolds in BTE is anticipated.

## Data Availability

Not applicable.

## References

[1] Amini A.R., Laurencin C.T., Nukavarapu S.P. (2012). Bone tissue engineering: Recent advances and challenges. Crit. Rev. Biomed. Eng..

[2] El-Rashidy A.A., Roether J.A., Harhaus L., Kneser U., Boccaccini A.R. (2017). Regenerating bone with bioactive glass scaffolds: A review of in vivo studies in bone defect models. Acta Biomater..

[3] Shen X., Zhang Y., Gu Y., Xu Y., Liu Y., Li B., Chen L. (2016). Sequential and sustained release of SDF-1 and BMP-2 from silk fibroin-nanohydroxyapatite scaffold for the enhancement of bone regeneration. Biomaterials.

[4] Tang D., Tare R.S., Yang L.-Y., Williams D.F., Ou K.-L., Oreffo R.O.C. (2016). Biofabrication of bone tissue: Approaches, challenges and translation for bone regeneration. Biomaterials.

[5] Khaled E.G., Saleh M., Hindocha S., Griffin M., Khan W.S. (2011). Tissue engineering for bone production- stem cells, gene therapy and scaffolds. Open Orthop. J..

[6] Sivakumar P.M., Yetisgin A.A., Sahin S.B., Demir E., Cetinel S. (2022). Bone tissue engineering: Anionic polysaccharides as promising scaffolds. Carbohydr. Polym..

[7] Qu M., Wang C., Zhou X., Libanori A., Jiang X., Xu W., Zhu S., Chen Q., Sun W., Khademhosseini A. (2021). Multi-Dimensional Printing for Bone Tissue Engineering. Adv. Healthc. Mater..

[8] Howard D., Buttery L.D., Shakesheff K.M., Roberts S.J. (2008). Tissue engineering: Strategies, stem cells and scaffolds. J. Anat..

[9] Song W., Muthana M., Mukherjee J., Falconer R.J., Biggs C.A., Zhao X. (2017). Magnetic-Silk Core-Shell Nanoparticles as Potential Carriers for Targeted Delivery of Curcumin into Human Breast Cancer Cells. ACS Biomater. Sci. Eng..

[10] Pham D.T., Tiyaboonchai W. (2020). Fibroin nanoparticles: A promising drug delivery system. Drug Deliv..

[11] Zheng H., Zuo B. (2021). Functional silk fibroin hydrogels: Preparation, properties and applications. J. Mater. Chem. B.

[12] Wei W., Liu J., Peng Z., Liang M., Wang Y., Wang X. (2020). Gellable silk fibroin-polyethylene sponge for hemostasis. Artif. Cells Nanomed. Biotechnol..

[13] Farokhi M., Mottaghitalab F., Reis R.L., Ramakrishna S., Kundu S.C. (2020). Functionalized silk fibroin nanofibers as drug carriers: Advantages and challenges. J. Control. Release.

[14] Agostinacchio F., Mu X., Dirè S., Motta A., Kaplan D.L. (2021). In Situ 3D Printing: Opportunities with Silk Inks. Trends Biotechnol..

[15] Saleem M., Rasheed S., Yougen C. (2020). Silk fibroin/hydroxyapatite scaffold: A highly compatible material for bone regeneration. Sci. Technol. Adv. Mater..

[16] Du X., Wei D., Huang L., Zhu M., Zhang Y., Zhu Y. (2019). 3D printing of mesoporous bioactive glass/silk fibroin composite scaffolds for bone tissue engineering. Mater. Sci. Eng. C Mater. Biol. Appl..

[17] Deininger C., Wagner A., Heimel P., Salzer E., Vila X.M., Weißenbacher N., Grillari J., Redl H., Wichlas F., Freude T. (2021). Enhanced BMP-2-Mediated Bone Repair Using an Anisotropic Silk Fibroin Scaffold Coated with Bone-like Apatite. Int. J. Mol. Sci..

[18] Wang B., Yuan S., Xin W., Chen Y., Fu Q., Li L., Jiao Y. (2021). Synergic adhesive chemistry-based fabrication of BMP-2 immobilized silk fibroin hydrogel functionalized with hybrid nanomaterial to augment osteogenic differentiation of rBMSCs for bone defect repair. Int. J. Biol. Macromol..

[19] Kundu B., Rajkhowa R., Kundu S.C., Wang X. (2013). Silk fibroin biomaterials for tissue regenerations. Adv. Drug Deliv. Rev..

[20] Farokhi M., Mottaghitalab F., Samani S., Shokrgozar M.A., Kundu S.C., Reis R.L., Fatahi Y., Kaplan D.L. (2018). Silk fibroin/hydroxyapatite composites for bone tissue engineering. Biotechnol. Adv..

[21] Melke J., Midha S., Ghosh S., Ito K., Hofmann S. (2016). Silk fibroin as biomaterial for bone tissue engineering. Acta Biomater..

[22] Zhou Z., Cui J., Wu S., Geng Z., Su J. (2022). Silk fibroin-based biomaterials for cartilage/osteochondral repair. Theranostics.

[23] Mottaghitalab F., Hosseinkhani H., Shokrgozar M.A., Mao C., Yang M., Farokhi M. (2015). Silk as a potential candidate for bone tissue engineering. J. Control. Release.

[24] Kasoju N., Bora U. (2012). Silk fibroin in tissue engineering. Adv. Healthc. Mater..

[25] Hao Z., Li H., Wang Y., Hu Y., Chen T., Zhang S., Guo X., Cai L., Li J. (2022). Supramolecular Peptide Nanofiber Hydrogels for Bone Tissue Engineering: From Multihierarchical Fabrications to Comprehensive Applications. Adv. Sci..

[26] Clarke B. (2008). Normal bone anatomy and physiology. Clin. J. Am. Soc. Nephrol..

[27] Guo L., Liang Z., Yang L., Du W., Yu T., Tang H., Li C., Qiu H. (2021). The role of natural polymers in bone tissue engineering. J. Control. Release.

[28] Singh S., Bray T.J.P., Hall-Craggs M.A. (2018). Quantifying bone structure, micro-architecture, and pathophysiology with MRI. Clin. Radiol..

[29] Liu Y., Luo D., Wang T. (2016). Hierarchical Structures of Bone and Bioinspired Bone Tissue Engineering. Small.

[30] Liu X., Wu Y., Zhao X., Wang Z. (2021). Fabrication and applications of bioactive chitosan-based organic-inorganic hybrid materials: A review. Carbohydr. Polym..

[31] Reznikov N., Shahar R., Weiner S. (2014). Bone hierarchical structure in three dimensions. Acta Biomater..

[32] Fang B., Qiu P., Xia C., Cai D., Zhao C., Chen Y., Wang H., Liu S., Cheng H., Tang Z. (2021). Extracellular matrix scaffold crosslinked with vancomycin for multifunctional antibacterial bone infection therapy. Biomaterials.

[33] Zhu L., Luo D., Liu Y. (2020). Effect of the nano/microscale structure of biomaterial scaffolds on bone regeneration. Int. J. Oral Sci..

[34] Zhang X., Chen X., Hong H., Hu R., Liu J., Liu C. (2022). Decellularized extracellular matrix scaffolds: Recent trends and emerging strategies in tissue engineering. Bioact. Mater..

[35] Omenetto F.G., Kaplan D.L. (2010). New opportunities for an ancient material. Science.

[36] Andersson M., Johansson J., Rising A. (2016). Silk Spinning in Silkworms and Spiders. Int. J. Mol. Sci..

[37] Johari N., Khodaei A., Samadikuchaksaraei A., Reis R.L., Kundu S.C., Moroni L. (2022). Ancient fibrous biomaterials from silkworm protein fibroin and spider silk blends: Biomechanical patterns. Acta Biomater..

[38] Solomun J.I., Totten J.D., Wongpinyochit T., Florence A.J., Seib F.P. (2020). Manual Versus Microfluidic-Assisted Nanoparticle Manufacture: Impact of Silk Fibroin Stock on Nanoparticle Characteristics. ACS Biomater. Sci. Eng..

[39] Ding Z., Cheng W., Mia M.S., Lu Q. (2021). Silk Biomaterials for Bone Tissue Engineering. Macromol. Biosci..

[40] Grabska-Zielińska S., Sionkowska A. (2021). How to Improve Physico-Chemical Properties of Silk Fibroin Materials for Biomedical Applications?-Blending and Cross-Linking of Silk Fibroin-A Review. Materials.

[41] Zou S., Yao X., Shao H., Reis R.L., Kundu S.C., Zhang Y. (2022). Nonmulberry silk fibroin-based biomaterials: Impact on cell behavior regulation and tissue regeneration. Acta Biomater..

[42] Zhou C.Z., Confalonieri F., Medina N., Zivanovic Y., Esnault C., Yang T., Jacquet M., Janin J., Duguet M., Perasso R. (2000). Fine organization of Bombyx mori fibroin heavy chain gene. Nucleic Acids Res..

[43] Zabelina V., Takasu Y., Sehadova H., Yonemura N., Nakajima K., Sezutsu H., Sery M., Zurovec M., Sehnal F., Tamura T. (2021). Mutation in Bombyx mori fibrohexamerin (P25) gene causes reorganization of rough endoplasmic reticulum in posterior silk gland cells and alters morphology of fibroin secretory globules in the silk gland lumen. Insect Biochem. Mol. Biol..

[44] Asakura T. (2021). Structure of Silk I (Silk Fibroin before Spinning) -Type II β-Turn, Not α-Helix. Molecules.

[45] Wang H.-Y., Zhang Y.-Q., Wei Z.-G. (2021). Dissolution and processing of silk fibroin for materials science. Crit. Rev. Biotechnol..

[46] Zhao Y., Zhu Z.S., Guan J., Wu S.J. (2021). Processing, mechanical properties and bio-applications of silk fibroin-based high-strength hydrogels. Acta Biomater..

[47] Wongpinyochit T., Johnston B.F., Seib F.P. (2018). Degradation Behavior of Silk Nanoparticles-Enzyme Responsiveness. ACS Biomater. Sci. Eng..

[48] DeBari M.K., King C.I., Altgold T.A., Abbott R.D. (2021). Silk Fibroin as a Green Material. ACS Biomater. Sci. Eng..

[49] Ode Boni B.O., Bakadia B.M., Osi A.R., Shi Z., Chen H., Gauthier M., Yang G. (2022). Immune Response to Silk Sericin-Fibroin Composites: Potential Immunogenic Elements and Alternatives for Immunomodulation. Macromol. Biosci..

[50] Rockwood D.N., Preda R.C., Yücel T., Wang X., Lovett M.L., Kaplan D.L. (2011). Materials fabrication from Bombyx mori silk fibroin. Nat. Protoc..

[51] Panilaitis B., Altman G.H., Chen J., Jin H.J., Karageorgiou V., Kaplan D.L. (2003). Macrophage responses to silk. Biomaterials.

[52] Kook G., Jeong S., Kim S.H., Kim M.K., Lee S., Cho I.-J., Choi N., Lee H.J. (2019). Wafer-Scale Multilayer Fabrication for Silk Fibroin-Based Microelectronics. ACS Appl. Mater. Interfaces.

[53] Choi J.H., Kim D.K., Song J.E., Oliveira J.M., Reis R.L., Khang G. (2018). Silk Fibroin-Based Scaffold for Bone Tissue Engineering. Adv. Exp. Med. Biol..

[54] Kwak H.W., Ju J.E., Shin M., Holland C., Lee K.H. (2017). Sericin Promotes Fibroin Silk I Stabilization Across a Phase-Separation. Biomacromolecules.

[55] Vepari C., Kaplan D.L. (2007). Silk as a Biomaterial. Prog. Polym. Sci..

[56] DeBari M.K., Abbott R.D. (2019). Microscopic considerations for optimizing silk biomaterials. Wiley Interdiscip. Rev. Nanomed. Nanobiotechnol..

[57] Nguyen T.P., Nguyen Q.V., Nguyen V.-H., Le T.-H., Huynh V.Q.N., Vo D.-V.N., Trinh Q.T., Kim S.Y., Le Q.V. (2019). Silk Fibroin-Based Biomaterials for Biomedical Applications: A Review. Polymers.

[58] Bucciarelli A., Greco G., Corridori I., Pugno N.M., Motta A. (2021). A Design of Experiment Rational Optimization of the Degumming Process and Its Impact on the Silk Fibroin Properties. ACS Biomater. Sci. Eng..

[59] Ha S.-W., Tonelli A.E., Hudson S.M. (2005). Structural studies of Bombyx mori silk fibroin during regeneration from solutions and wet fiber spinning. Biomacromolecules.

[60] Hong H., Lee O.J., Lee Y.J., Lee J.S., Ajiteru O., Lee H., Suh Y.J., Sultan M.T., Kim S.H., Park C.H. (2020). Cytocompatibility of Modified Silk Fibroin with Glycidyl Methacrylate for Tissue Engineering and Biomedical Applications. Biomolecules.

[61] Zhao M., Qi Z., Tao X., Newkirk C., Hu X., Lu S. (2021). Chemical, Thermal, Time, and Enzymatic Stability of Silk Materials with Silk I Structure. Int. J. Mol. Sci..

[62] Sheng R., Chen J., Wang H., Luo Y., Liu J., Chen Z., Mo Q., Chi J., Ling C., Tan X. (2022). Nanosilicate-Reinforced Silk Fibroin Hydrogel for Endogenous Regeneration of Both Cartilage and Subchondral Bone. Adv. Healthc. Mater..

[63] Zhou Y., Hu Y., Uemura M., Xia L., Yu X., Xu Y. (2022). Fabrication and Effect of Strontium-Substituted Calcium Silicate/Silk Fibroin on Bone Regeneration and. Front. Bioeng. Biotechnol..

[64] Chen F., Porter D., Vollrath F. (2012). Morphology and structure of silkworm cocoons. Mater. Sci. Eng. C.

[65] Meinel L., Hofmann S., Karageorgiou V., Kirker-Head C., McCool J., Gronowicz G., Zichner L., Langer R., Vunjak-Novakovic G., Kaplan D.L. (2005). The inflammatory responses to silk films in vitro and in vivo. Biomaterials.

[66] Santin M., Motta A., Freddi G., Cannas M. (1999). In vitro evaluation of the inflammatory potential of the silk fibroin. J. Biomed. Mater. Res..

[67] Minoura N., Aiba S., Higuchi M., Gotoh Y., Tsukada M., Imai Y. (1995). Attachment and growth of fibroblast cells on silk fibroin. Biochem. Biophys. Res. Commun..

[68] Fan H., Liu H., Toh S.L., Goh J.C.H. (2009). Anterior cruciate ligament regeneration using mesenchymal stem cells and silk scaffold in large animal model. Biomaterials.

[69] Jo Y.-Y., Kim S.-G., Kwon K.-J., Kweon H., Chae W.-S., Yang W.-G., Lee E.-Y., Seok H. (2017). Silk Fibroin-Alginate-Hydroxyapatite Composite Particles in Bone Tissue Engineering Applications In Vivo. Int. J. Mol. Sci..

[70] Luetchford K.A., Chaudhuri J.B., De Bank P.A. (2020). Silk fibroin/gelatin microcarriers as scaffolds for bone tissue engineering. Mater. Sci. Eng. C Mater. Biol. Appl..

[71] Kirillova A., Yeazel T.R., Asheghali D., Petersen S.R., Dort S., Gall K., Becker M.L. (2021). Fabrication of Biomedical Scaffolds Using Biodegradable Polymers. Chem. Rev..

[72] Cao Y., Wang B. (2009). Biodegradation of silk biomaterials. Int. J. Mol. Sci..

[73] Numata K., Cebe P., Kaplan D.L. (2010). Mechanism of enzymatic degradation of beta-sheet crystals. Biomaterials.

[74] Park S.-H., Gil E.S., Kim H.J., Lee K., Kaplan D.L. (2010). Relationships between degradability of silk scaffolds and osteogenesis. Biomaterials.

[75] Horan R.L., Bramono D.S., Stanley J.R.L., Simmons Q., Chen J., Boepple H.E., Altman G.H. (2009). Biological and biomechanical assessment of a long-term bioresorbable silk-derived surgical mesh in an abdominal body wall defect model. Hernia J. Hernias Abdom. Wall Surg..

[76] Lu Q., Hu X., Wang X., Kluge J.A., Lu S., Cebe P., Kaplan D.L. (2010). Water-insoluble silk films with silk I structure. Acta Biomater..

[77] Sengupta S., Park S.-H., Seok G.E., Patel A., Numata K., Lu C.-L., Kaplan D.L. (2010). Quantifying osteogenic cell degradation of silk biomaterials. Biomacromolecules.

[78] Wang Y., Rudym D.D., Walsh A., Abrahamsen L., Kim H.-J., Kim H.S., Kirker-Head C., Kaplan D.L. (2008). In vivo degradation of three-dimensional silk fibroin scaffolds. Biomaterials.

[79] Umuhoza D., Yang F., Long D., Hao Z., Dai J., Zhao A. (2020). Strategies for Tuning the Biodegradation of Silk Fibroin-Based Materials for Tissue Engineering Applications. ACS Biomater. Sci. Eng..

[80] Horan R.L., Toponarski I., Boepple H.E., Weitzel P.P., Richmond J.C., Altman G.H. (2009). Design and characterization of a scaffold for anterior cruciate ligament engineering. J. Knee Surg..

[81] Diab T., Pritchard E.M., Uhrig B.A., Boerckel J.D., Kaplan D.L., Guldberg R.E. (2012). A silk hydrogel-based delivery system of bone morphogenetic protein for the treatment of large bone defects. J. Mech. Behav. Biomed. Mater..

[82] Kim J.H., Park C.H., Lee O.-J., Lee J.-M., Kim J.W., Park Y.H., Ki C.S. (2012). Preparation and in vivo degradation of controlled biodegradability of electrospun silk fibroin nanofiber mats. J. Biomed. Mater. Res. A.

[83] Bhattacharjee M., Schultz-Thater E., Trella E., Miot S., Das S., Loparic M., Ray A.R., Martin I., Spagnoli G.C., Ghosh S. (2013). The role of 3D structure and protein conformation on the innate and adaptive immune responses to silk-based biomaterials. Biomaterials.

[84] He Y., Yang X., Yuan Z., Shen X., Xu K., Lin C., Tao B., Li K., Chen M., Hu Y. (2019). Regulation of MSC and macrophage functions in bone healing by peptide LL-37-loaded silk fibroin nanoparticles on a titanium surface. Biomater. Sci..

[85] Naskar D., Nayak S., Dey T., Kundu S.C. (2014). Non-mulberry silk fibroin influence osteogenesis and osteoblast-macrophage cross talk on titanium based surface. Sci. Rep..

[86] Um I.C., Kweon H.Y., Park Y.H., Hudson S. (2001). Structural characteristics and properties of the regenerated silk fibroin prepared from formic acid. Int. J. Biol. Macromol..

[87] Sun W., Gregory D.A., Tomeh M.A., Zhao X. (2021). Silk Fibroin as a Functional Biomaterial for Tissue Engineering. Int. J. Mol. Sci..

[88] Lawrence B.D., Pan Z., Weber M.D., Kaplan D.L., Rosenblatt M.I. (2012). Silk film culture system for in vitro analysis and biomaterial design. J. Vis. Exp. JoVE.

[89] Hu X., Shmelev K., Sun L., Gil E.-S., Park S.-H., Cebe P., Kaplan D.L. (2011). Regulation of silk material structure by temperature-controlled water vapor annealing. Biomacromolecules.

[90] Wang C., Fang H., Qi X., Hang C., Sun Y., Peng Z., Wei W., Wang Y. (2019). Silk fibroin film-coated MgZnCa alloy with enhanced in vitro and in vivo performance prepared using surface activation. Acta Biomater..

[91] Wenk E., Wandrey A.J., Merkle H.P., Meinel L. (2008). Silk fibroin spheres as a platform for controlled drug delivery. J. Control. Release.

[92] Cao Z., Chen X., Yao J., Huang L., Shao Z. (2007). The preparation of regenerated silk fibroin microspheres. Soft Matter.

[93] Zhao Z., Li Y., Xie M.-B. (2015). Silk fibroin-based nanoparticles for drug delivery. Int. J. Mol. Sci..

[94] Doane T.L., Burda C. (2012). The unique role of nanoparticles in nanomedicine: Imaging, drug delivery and therapy. Chem. Soc. Rev..

[95] Florczak A., Grzechowiak I., Deptuch T., Kucharczyk K., Kaminska A., Dams-Kozlowska H. (2020). Silk Particles as Carriers of Therapeutic Molecules for Cancer Treatment. Materials.

[96] Subia B., Chandra S., Talukdar S., Kundu S.C. (2014). Folate conjugated silk fibroin nanocarriers for targeted drug delivery. Integr. Biol. Quant. Biosci. Nano Macro.

[97] Mao B., Liu C., Zheng W., Li X., Ge R., Shen H., Guo X., Lian Q., Shen X., Li C. (2018). Cyclic cRGDfk peptide and Chlorin e6 functionalized silk fibroin nanoparticles for targeted drug delivery and photodynamic therapy. Biomaterials.

[98] Mottaghitalab F., Kiani M., Farokhi M., Kundu S.C., Reis R.L., Gholami M., Bardania H., Dinarvand R., Geramifar P., Beiki D. (2017). Targeted Delivery System Based on Gemcitabine-Loaded Silk Fibroin Nanoparticles for Lung Cancer Therapy. ACS Appl. Mater. Interfaces.

[99] Bian X., Wu P., Sha H., Qian H., Wang Q., Cheng L., Yang Y., Yang M., Liu B. (2016). Anti-EGFR-iRGD recombinant protein conjugated silk fibroin nanoparticles for enhanced tumor targeting and antitumor efficiency. Onco. Targets Ther..

[100] Gou S., Huang Y., Wan Y., Ma Y., Zhou X., Tong X., Huang J., Kang Y., Pan G., Dai F. (2019). Multi-bioresponsive silk fibroin-based nanoparticles with on-demand cytoplasmic drug release capacity for CD44-targeted alleviation of ulcerative colitis. Biomaterials.

[101] Rodriguez-Nogales A., Algieri F., De Matteis L., Lozano-Perez A.A., Garrido-Mesa J., Vezza T., de la Fuente J.M., Cenis J.L., Gálvez J., Rodriguez-Cabezas M.E. (2016). Intestinal anti-inflammatory effects of RGD-functionalized silk fibroin nanoparticles in trinitrobenzenesulfonic acid-induced experimental colitis in rats. Int. J. Nanomed..

[102] Bari E., Serra M., Paolillo M., Bernardi E., Tengattini S., Piccinini F., Lanni C., Sorlini M., Bisbano G., Calleri E. (2021). Silk Fibroin Nanoparticle Functionalization with Arg-Gly-Asp Cyclopentapeptide Promotes Active Targeting for Tumor Site-Specific Delivery. Cancers.

[103] Bhardwaj N., Chakraborty S., Kundu S.C. (2011). Freeze-gelled silk fibroin protein scaffolds for potential applications in soft tissue engineering. Int. J. Biol. Macromol..

[104] Calderón-Colón X., Xia Z., Breidenich J.L., Mulreany D.G., Guo Q., Uy O.M., Tiffany J.E., Freund D.E., McCally R.L., Schein O.D. (2012). Structure and properties of collagen vitrigel membranes for ocular repair and regeneration applications. Biomaterials.

[105] Sahoo J.K., Choi J., Hasturk O., Laubach I., Descoteaux M.L., Mosurkal S., Wang B., Zhang N., Kaplan D.L. (2020). Silk degumming time controls horseradish peroxidase-catalyzed hydrogel properties. Biomater. Sci..

[106] McGill M., Grant J.M., Kaplan D.L. (2020). Enzyme-Mediated Conjugation of Peptides to Silk Fibroin for Facile Hydrogel Functionalization. Ann. Biomed. Eng..

[107] Wang X., Kluge J.A., Leisk G.G., Kaplan D.L. (2008). Sonication-induced gelation of silk fibroin for cell encapsulation. Biomaterials.

[108] Catoira M.C., Fusaro L., Di Francesco D., Ramella M., Boccafoschi F. (2019). Overview of natural hydrogels for regenerative medicine applications. J. Mater. Sci. Mater. Med..

[109] Matsumoto A., Chen J., Collette A.L., Kim U.-J., Altman G.H., Cebe P., Kaplan D.L. (2006). Mechanisms of silk fibroin sol-gel transitions. J. Phys. Chem. B.

[110] Kim U.-J., Park J., Li C., Jin H.-J., Valluzzi R., Kaplan D.L. (2004). Structure and properties of silk hydrogels. Biomacromolecules.

[111] Kundu J., Poole-Warren L.A., Martens P., Kundu S.C. (2012). Silk fibroin/poly(vinyl alcohol) photocrosslinked hydrogels for delivery of macromolecular drugs. Acta Biomater..

[112] Rnjak-Kovacina J., Wray L.S., Burke K.A., Torregrosa T., Golinski J.M., Huang W., Kaplan D.L. (2015). Lyophilized Silk Sponges: A Versatile Biomaterial Platform for Soft Tissue Engineering. ACS Biomater. Sci. Eng..

[113] Wang L., Fang M., Xia Y., Hou J., Nan X., Zhao B., Wang X. (2020). Preparation and biological properties of silk fibroin/nano-hydroxyapatite/graphene oxide scaffolds with an oriented channel-like structure. RSC Adv..

[114] Bicho D., Canadas R.F., Gonçalves C., Pina S., Reis R.L., Oliveira J.M. (2021). Porous aligned ZnSr-doped β-TCP/silk fibroin scaffolds using ice-templating method for bone tissue engineering applications. J. Biomater. Sci. Polym. Ed..

[115] Font Tellado S., Bonani W., Balmayor E.R., Foehr P., Motta A., Migliaresi C., van Griensven M. (2017). (*) Fabrication and Characterization of Biphasic Silk Fibroin Scaffolds for Tendon/Ligament-to-Bone Tissue Engineering. Tissue Eng. Part A.

[116] Nazarov R., Jin H.-J., Kaplan D.L. (2004). Porous 3-D scaffolds from regenerated silk fibroin. Biomacromolecules.

[117] Yin Y., Xiong J. (2018). Finite Element Analysis of Electrospun Nanofibrous Mats under Biaxial Tension. Nanomaterials.

[118] Jacobsen M.M., Li D., Gyune Rim N., Backman D., Smith M.L., Wong J.Y. (2017). Silk-fibronectin protein alloy fibres support cell adhesion and viability as a high strength, matrix fibre analogue. Sci. Rep..

[119] Zhang C., Zhang Y., Shao H., Hu X. (2016). Hybrid Silk Fibers Dry-Spun from Regenerated Silk Fibroin/Graphene Oxide Aqueous Solutions. ACS Appl. Mater. Interfaces.

[120] Xue J., Wu T., Dai Y., Xia Y. (2019). Electrospinning and Electrospun Nanofibers: Methods, Materials, and Applications. Chem. Rev..

[121] Zhang X., Reagan M.R., Kaplan D.L. (2009). Electrospun silk biomaterial scaffolds for regenerative medicine. Adv. Drug Deliv. Rev..

[122] Park B.K., Um I.C. (2018). Effect of molecular weight on electro-spinning performance of regenerated silk. Int. J. Biol. Macromol..

[123] Mao Y., Zhao Y., Guan J., Guan J., Ye T., Chen Y., Zhu Y., Zhou P., Cui W. (2020). Electrospun fibers: An innovative delivery method for the treatment of bone diseases. Expert Opin. Drug Deliv..

[124] Gupta S., Prasad P., Roy A., Alam M.M., Ahmed I., Bit A. (2022). Metallic ion-based graphene oxide functionalized silk fibroin-based dressing promotes wound healing via improved bactericidal outcomes and faster re-epithelization. Biomed. Mater..

[125] Lan D., Liu Z., Zhou J., Xu M., Li Z., Dai F. (2022). Preparation and characterization of silk fibroin/polyethylene oxide nanofiber membranes with antibacterial activity. J. Biomed. Mater. Res. A.

[126] Sharifi E., Azami M., Kajbafzadeh A.-M., Moztarzadeh F., Faridi-Majidi R., Shamousi A., Karimi R., Ai J. (2016). Preparation of a biomimetic composite scaffold from gelatin/collagen and bioactive glass fibers for bone tissue engineering. Mater. Sci. Eng. C Mater. Biol. Appl..

[127] Chakraborty J., Mu X., Pramanick A., Kaplan D.L., Ghosh S. (2022). Recent advances in bioprinting using silk protein-based bioinks. Biomaterials.

[128] Matai I., Kaur G., Seyedsalehi A., McClinton A., Laurencin C.T. (2020). Progress in 3D bioprinting technology for tissue/organ regenerative engineering. Biomaterials.

[129] Wang Q., Han G., Yan S., Zhang Q. (2019). 3D Printing of Silk Fibroin for Biomedical Applications. Materials.

[130] DeSimone E., Schacht K., Jungst T., Groll J., Scheibel T. (2015). Biofabrication of 3D constructs: Fabrication technologies and spider silk proteins as bioinks. Pure Appl. Chem..

[131] Suntivich R., Drachuk I., Calabrese R., Kaplan D.L., Tsukruk V.V. (2014). Inkjet printing of silk nest arrays for cell hosting. Biomacromolecules.

[132] Gasperini L., Mano J.F., Reis R.L. (2014). Natural polymers for the microencapsulation of cells. J. R. Soc. Interface.

[133] Włodarczyk-Biegun M.K., Del Campo A. (2017). 3D bioprinting of structural proteins. Biomaterials.

[134] Gopinathan J., Noh I. (2018). Recent trends in bioinks for 3D printing. Biomater. Res..

[135] Costa J.B., Silva-Correia J., Oliveira J.M., Reis R.L. (2017). Fast Setting Silk Fibroin Bioink for Bioprinting of Patient-Specific Memory-Shape Implants. Adv. Healthc. Mater..

[136] Kim S.H., Yeon Y.K., Lee J.M., Chao J.R., Lee Y.J., Seo Y.B., Sultan M.T., Lee O.J., Lee J.S., Yoon S.-I. (2018). Precisely printable and biocompatible silk fibroin bioink for digital light processing 3D printing. Nat. Commun..

[137] Rajput M., Mondal P., Yadav P., Chatterjee K. (2022). Light-based 3D bioprinting of bone tissue scaffolds with tunable mechanical properties and architecture from photocurable silk fibroin. Int. J. Biol. Macromol..

[138] Lee Y.J., Lee J.S., Ajiteru O., Lee O.J., Lee J.S., Lee H., Kim S.W., Park J.W., Kim K.Y., Choi K.Y. (2022). Biocompatible fluorescent silk fibroin bioink for digital light processing 3D printing. Int. J. Biol. Macromol..

[139] Li Y., Liu Z., Tang Y., Fan Q., Feng W., Luo C., Dai G., Ge Z., Zhang J., Zou G. (2020). Three-dimensional silk fibroin scaffolds enhance the bone formation and angiogenic differentiation of human amniotic mesenchymal stem cells: A biocompatibility analysis. Acta Biochim. Biophys. Sin..

[140] Chen B.Q., Kankala R.K., Chen A.Z., Yang D.Z., Cheng X.X., Jiang N.N., Zhu K., Wang S.B. (2017). Investigation of silk fibroin nanoparticle-decorated poly(l-lactic acid) composite scaffolds for osteoblast growth and differentiation. Int. J. Nanomed..

[141] Kim M.H., Kim B.S., Lee J., Cho D., Kwon O.H., Park W.H. (2017). Silk fibroin/hydroxyapatite composite hydrogel induced by gamma-ray irradiation for bone tissue engineering. Biomater. Res..

[142] Jia X., Zhou J., Ning J., Li M., Yao Y., Wang X., Jian Y., Zhao K. (2022). The polycaprolactone/silk fibroin/carbonate hydroxyapatite electrospun scaffold promotes bone reconstruction by regulating the polarization of macrophages. Regen. Biomater..

[143] Del Bianco L., Spizzo F., Yang Y., Greco G., Gatto M.L., Barucca G., Pugno N.M., Motta A. (2022). Silk fibroin films with embedded magnetic nanoparticles: Evaluation of the magneto-mechanical stimulation effect on osteogenic differentiation of stem cells. Nanoscale.

[144] Wu J., Cao L., Liu Y., Zheng A., Jiao D., Zeng D., Wang X., Kaplan D.L., Jiang X. (2019). Functionalization of Silk Fibroin Electrospun Scaffolds via BMSC Affinity Peptide Grafting through Oxidative Self-Polymerization of Dopamine for Bone Regeneration. ACS Appl. Mater. Interfaces.

[145] Moses J.C., Dey S., Bandyopadhyay A., Agarwala M., Mandal B.B. (2022). Silk-Based Bioengineered Diaphyseal Cortical Bone Unit Enclosing an Implantable Bone Marrow toward Atrophic Nonunion Grafting. Adv. Healthc. Mater..

[146] Ou L., Lan Y., Feng Z., Feng L., Yang J., Liu Y., Bian L., Tan J., Lai R., Guo R. (2019). Functionalization of SF/HAP Scaffold with GO-PEI-miRNA inhibitor Complexes to Enhance Bone Regeneration through Activating Transcription Factor 4. Theranostics.

[147] Chen G., Deng C., Li Y.-P. (2012). TGF-β and BMP signaling in osteoblast differentiation and bone formation. Int. J. Biol. Sci..

[148] Koolen P.G.L., Haas D., Kim K., Fox S., Ibrahim A.M.S., Kim P., Kaplan D.L., Lin S.J. (2016). Increased Osteoid Formation in BMP-2-Loaded Silk-Based Screws. Plast. Reconstr. Surg..

[149] Bessa P.C., Balmayor E.R., Hartinger J., Zanoni G., Dopler D., Meinl A., Banerjee A., Casal M., Redl H., Reis R.L. (2010). Silk fibroin microparticles as carriers for delivery of human recombinant bone morphogenetic protein-2: In vitro and in vivo bioactivity. Tissue Eng. Part C Methods.

[150] Shi P., Abbah S.A., Saran K., Zhang Y., Li J., Wong H.-K., Goh J.C.H. (2013). Silk fibroin-based complex particles with bioactive encrustation for bone morphogenetic protein 2 delivery. Biomacromolecules.

[151] Bessa P.C., Balmayor E.R., Azevedo H.S., Nürnberger S., Casal M., van Griensven M., Reis R.L., Redl H. (2010). Silk fibroin microparticles as carriers for delivery of human recombinant BMPs. Physical characterization and drug release. J. Tissue Eng. Regen. Med..

[152] James A.W., LaChaud G., Shen J., Asatrian G., Nguyen V., Zhang X., Ting K., Soo C. (2016). A Review of the Clinical Side Effects of Bone Morphogenetic Protein-2. Tissue Eng. Part B Rev..

[153] Fuerkaiti S.N., Çakmak A.S., Karaaslan C., Gümüşderelioğlu M. (2022). Enhanced osteogenic effect in reduced BMP-2 doses with siNoggin transfected pre-osteoblasts in 3D silk scaffolds. Int. J. Pharm..

[154] Song Y., Wu H., Gao Y., Li J., Lin K., Liu B., Lei X., Cheng P., Zhang S., Wang Y. (2020). Zinc Silicate/Nano-Hydroxyapatite/Collagen Scaffolds Promote Angiogenesis and Bone Regeneration via the p38 MAPK Pathway in Activated Monocytes. ACS Appl. Mater. Interfaces.

[155] Kanczler J.M., Oreffo R.O.C. (2008). Osteogenesis and angiogenesis: The potential for engineering bone. Eur. Cells Mater..

[156] Zhang D., Cao N., Zhou S., Chen Z., Zhang X., Zhu W. (2018). The enhanced angiogenesis effect of VEGF-silk fibroin nanospheres-BAMG scaffold composited with adipose derived stem cells in a rabbit model. RSC Adv..

[157] Hassani Besheli N., Damoogh S., Zafar B., Mottaghitalab F., Motasadizadeh H., Rezaei F., Shokrgozar M.A., Farokhi M. (2018). Preparation of a Codelivery System Based on Vancomycin/Silk Scaffold Containing Silk Nanoparticle Loaded VEGF. ACS Biomater. Sci. Eng..

[158] Bai S., Zhang X., Lu Q., Sheng W., Liu L., Dong B., Kaplan D.L., Zhu H. (2014). Reversible hydrogel-solution system of silk with high beta-sheet content. Biomacromolecules.

[159] Zhang W., Wang X., Wang S., Zhao J., Xu L., Zhu C., Zeng D., Chen J., Zhang Z., Kaplan D.L. (2011). The use of injectable sonication-induced silk hydrogel for VEGF(165) and BMP-2 delivery for elevation of the maxillary sinus floor. Biomaterials.

[160] Wang Q., Zhang Y., Li B., Chen L. (2017). Controlled dual delivery of low doses of BMP-2 and VEGF in a silk fibroin-nanohydroxyapatite scaffold for vascularized bone regeneration. J. Mater. Chem. B.

[161] Zhu J., Xiong J., Ji W. (2022). A Systematic Review of Bone Marrow Stromal Cells and Periosteum-Derived Cells for Bone Regeneration. Tissue Eng. Part B Rev..

[162] Ye J.-H., Xu Y.-J., Gao J., Yan S.-G., Zhao J., Tu Q., Zhang J., Duan X.-J., Sommer C.A., Mostoslavsky G. (2011). Critical-size calvarial bone defects healing in a mouse model with silk scaffolds and SATB2-modified iPSCs. Biomaterials.

[163] Han Q., Yang P., Wu Y., Meng S., Sui L., Zhang L., Yu L., Tang Y., Jiang H., Xuan D. (2015). Epigenetically Modified Bone Marrow Stromal Cells in Silk Scaffolds Promote Craniofacial Bone Repair and Wound Healing. Tissue Eng. Part A.

[164] Jin H.-J., Chen J., Karageorgiou V., Altman G.H., Kaplan D.L. (2004). Human bone marrow stromal cell responses on electrospun silk fibroin mats. Biomaterials.

[165] Rockwood D.N., Gil E.S., Park S.-H., Kluge J.A., Grayson W., Bhumiratana S., Rajkhowa R., Wang X., Kim S.J., Vunjak-Novakovic G. (2011). Ingrowth of human mesenchymal stem cells into porous silk particle reinforced silk composite scaffolds: An in vitro study. Acta Biomater..

[166] Ai C., Liu L., Goh J.C.-H. (2021). Pore size modulates in vitro osteogenesis of bone marrow mesenchymal stem cells in fibronectin/gelatin coated silk fibroin scaffolds. Mater. Sci. Eng. C Mater. Biol. Appl..

[167] Li C., Vepari C., Jin H.-J., Kim H.J., Kaplan D.L. (2006). Electrospun silk-BMP-2 scaffolds for bone tissue engineering. Biomaterials.

[168] Karageorgiou V., Tomkins M., Fajardo R., Meinel L., Snyder B., Wade K., Chen J., Vunjak-Novakovic G., Kaplan D.L. (2006). Porous silk fibroin 3-D scaffolds for delivery of bone morphogenetic protein-2 in vitro and in vivo. J. Biomed. Mater. Res. A.

[169] Wang N., Liu X., Tang Z., Wei X., Dong H., Liu Y., Wu H., Wu Z., Li X., Ma X. (2022). Increased BMSC exosomal miR-140-3p alleviates bone degradation and promotes bone restoration by targeting Plxnb1 in diabetic rats. J. Nanobiotechnol..

[170] Li Z., Li Q., Tong K., Zhu J., Wang H., Chen B., Chen L. (2022). BMSC-derived exosomes promote tendon-bone healing after anterior cruciate ligament reconstruction by regulating M1/M2 macrophage polarization in rats. Stem Cell Res. Ther..

[171] Lei F., Li M., Lin T., Zhou H., Wang F., Su X. (2022). Treatment of inflammatory bone loss in periodontitis by stem cell-derived exosomes. Acta Biomater..

[172] Sun M., Li Q., Yu H., Cheng J., Wu N., Shi W., Zhao F., Shao Z., Meng Q., Chen H. (2022). Cryo-self-assembled silk fibroin sponge as a biodegradable platform for enzyme-responsive delivery of exosomes. Bioact. Mater..

